# The significance of the apelinergic system in doxorubicin-induced cardiotoxicity

**DOI:** 10.1007/s10741-024-10414-w

**Published:** 2024-07-11

**Authors:** Katarzyna Matusik, Katarzyna Kamińska, Aleksandra Sobiborowicz-Sadowska, Hubert Borzuta, Kasper Buczma, Agnieszka Cudnoch-Jędrzejewska

**Affiliations:** Department of Experimental and Clinical Physiology, Laboratory of Centre for Preclinical Research, Medical University of Warsaw, Warsaw, Poland

**Keywords:** Apelinergic system, Cancer, Cardiotoxicity, Doxorubicin

## Abstract

Cancer is the leading cause of death worldwide, and the number of cancer-related deaths is expected to increase. Common types of cancer include skin, breast, lung, prostate, and colorectal cancers. While clinical research has improved cancer therapies, these treatments often come with significant side effects such as chronic fatigue, hair loss, and nausea. In addition, cancer treatments can cause long-term cardiovascular complications. Doxorubicin (DOX) therapy is one example, which can lead to decreased left ventricle (LV) echocardiography (ECHO) parameters, increased oxidative stress in cellular level, and even cardiac fibrosis. The apelinergic system, specifically apelin and its receptor, together, has shown properties that could potentially protect the heart and mitigate the damages caused by DOX anti-cancer treatment. Studies have suggested that stimulating the apelinergic system may have therapeutic benefits for heart damage induced by DOX. Further research in chronic preclinical models is needed to confirm this hypothesis and understand the mechanism of action for the apelinergic system. This review aims to collect and present data on the effects of the apelinergic system on doxorubicin-induced cardiotoxicity.

## Introduction

Cancer is one of the most common causes of death worldwide and the number of cancer deaths continues to increase globally. According to World Cancer Research Fund International, about 10 million people died from cancer in 2020, and nearly 29 million cancer-related deaths are expected in 2040 [1]. Apart from skin cancers, breast, lung, prostate, and colorectal cancers are the most commonly diagnosed types of malignancies [2].

Advances in clinical research have increased the range of effective anti-cancer therapies in clinical practice, but their adverse effects are often a major challenge. Many patients experience serious side effects from anti-cancer drugs including the non-selective effects of chemotherapeutics. Among the most commonly reported side effects, due to the unselective action of antineoplastic agents, are chronic fatigue, alopecia, nausea, vomiting, oral mucositis, diarrhea, hematologic disturbances such as anemia or neutropenia, and lymphedema, as well as thromboembolic events [3, 4].

The aforementioned adverse effects are acute in nature and usually subside after the completion of the treatment. However, most significantly, oncologic therapy can also leave notable chronic cardiovascular complications behind, that can occur either during the treatment or many years afterward [5].

Due to individual variability, some patients are more likely to develop these harmful side effects. Predisposing factors for cardiovascular complications during and after treatment include less than a year of age or very advanced age [6], female sex [6, 7], trisomy 21, and African-American ancestry [8]. But most importantly, pre-existing heart disease shown in medical history and type of therapy used are considered the most significant risk factors for cardiovascular toxicity during anti-cancer therapy [9].

Also, comorbidities, especially metabolic diseases such as diabetes, and also obesity, endocrine disorders, and renal and pulmonary disease correlate with the occurrence of cardiac events [9]. Barry et al. highlight the importance of pre-existing cardiac risk factors such as hypertension, ischemia, arrythmias, and myocardial and valvular heart disease in the development of subsequent cardiotoxicity [9].

The most serious side effect of cancer therapy, apart from the death of the patient, is the cardiotoxicity that occurs as an effect of exposure to anti-cancer agents. Cardiotoxicity associated with oncologic therapy can manifest as coronary artery disease (CAD), cardiac arrhythmias, and conduction abnormalities, but the most frequent clinical manifestation of cardiotoxicity is dose-dependent cardiomyopathy. Until 2021, there was no uniform definition of cardiotoxicity related to cancer therapy. The common denominator, however, was the development of left ventricular systolic dysfunction (LVSD) with a reduction in left ventricular ejection fraction (LVEF).

In 2021, International Cardio-Oncology Society (IC-OS) published a standardized definition of termed cancer therapy-related cardiac dysfunction (CTRCD). According to Herrmann et al. [10], the following can be distinguished: symptomatic CTRCD associated with heart failure (HF) and structural abnormalities, impaired cardiac function, impaired myocardial perfusion and/or volume overload and asymptomatic CTRCD with assessment of LVEF unrelated to HF. There is a 3-point severity scale for asymptomatic CTRCD: (1) mild, when EF ≥ 50% with > 15% decrease in global longitudinal strain (GLS) from baseline or increase in previously tested troponin/natriuretic peptide levels; (2) moderate, when EF falls ≥ 40–49%; and (3) severe, when EF drops to < 40% [10].

There are two types of cardiotoxicity, according to Ewer and Lippman’s (2005) classification, type 1 and type 2 [11]. Type 1 cardiotoxicity is characterized by non-reversible myocardial damage caused by a cumulative dose of anti-cancer drugs, while type 2 is marked by dose-independent reversible myocardial damage [12].

Anthracyclines (ANT), a class of drugs first extracted from *Streptomyces bacterium*, are the prime example of drugs that cause type 1 of cardiotoxicity. These agents are also one of the most effective anti-cancer drugs ever developed and are highly effective against more types of cancer than any other class of chemotherapeutic agents [13].

Currently, among the ANT clinically used are doxorubicin (DOX), daunorubicin, epirubicin, idarubicin, and mitoxantrone. All known ANT have toxic effects on the myocardium [14].

Despite advances in the field of oncology, the search for the most effective drug/therapy acting against the development of cardiotoxicity induced by ANT still continues, regardless of the good results of treatment with dexrazoxane and liposomal forms of ANT. Unfortunately, despite continued screening, implementation of monitoring methods, and collaboration between oncologists and cardiologists, the number of patients with chemotherapy-related cardiotoxicity is increasing. Among the reasons for this phenomenon, it is worth mentioning that more patients are surviving their cancer and have a long life after chemotherapy; and therefore, it is possible to diagnose late cardiotoxicity. Accordingly, new substances/therapies are being sought to prevent or improve cardiovascular parameters such as LVSD with reduction in LVEF and maintain these parameters over the long term. Recently, the apelinergic system has received much attention in this aspect, due to its involvement in cardiovascular system regulation.

The presence of apelinergic system components—apelin (APL), elabela (ELA), and apelin receptor (APJ)—has been detected widely in cardiomyocytes, endothelium, and vascular smooth muscle. In the endothelium, APJ activation results in vasorelaxation due to the stimulation of nitric oxide (NO) release [15], whereas that in smooth muscle contributes to the vasoconstriction [17]. Moreover, APL and ELA were found to exert a positive inotropic effect on the heart [16] and to play an important role in the central regulation of the cardiovascular system [6]. Dysregulation of the apelinergic system has been studied inin vivo/ex vivo preclinical research of HF and myocardial infarction (MI) [18–20].

Downregulation of the apelinergic system in animal models has been shown to decrease exercise capacity under physiological stress, induce progressive HF, and show susceptibility to cardiac oxidative stress [21]. Cardioprotective action of the apelinergic system has been widely documented within the last few years. Treatment with APL and ELA reduced myocardial inflammation, limited myocardial injury, and significantly improved cardiorenal function in animal models. Administration of ELA limited the area of cardiac fibrosis in murine MI models with downregulated levels of profibrotic genes [22]. Such studies show the potential of the apelinergic system to become one of the possible pathways leading to the reduction of anthracycline-induced cardiotoxicity, especially the frequently used DOX.

Given the above, the purpose of this review is to assemble and demonstrate data on the effect of the apelinergic system on DOX-induced cardiotoxicity.

## Doxorubicin-induced cardiotoxicity

ANT were discovered more than 60 years ago [23]. These agents are used in the treatment of acute lymphoblastic and myeloblastic leukemia, breast cancer, Ewing’s sarcoma, and various types of lymphomas, along with Hodgkin’s lymphoma, in both children and adults [24]. Despite their high efficacy, ANT act non-selectively, causing damage not only to the cancer cells but also to the healthy cells, including cardiomyocytes. Those effects are associated with the occurrence of CTRCD, manifested by a wide range of left ventricle (LV) contraction functions [12].

The most frequently administered drug from the ANT group in a treatment of cancers mentioned above is DOX, also known as adriamycin, an effective anti-neoplastic agent obtained by aerobic fermentation from *Streptomyces peucetius* var *caesius* [25].

DOX has been used in cancer therapy for several decades. In the late 1970s, the effects of DOX on the cardiovascular system were determined to be cardiotoxic [24]. DOX-induced cardiotoxicity (DIC) is characterized by a different time of onset and a broad spectrum of symptoms [25].

Acute cardiotoxicity begins with changes at the cellular level in the form of myocardial cell injury and myocardial deformation connected with change in the length of the myocardial wall [26] developing in the first weeks of treatment. This is a rare type of cardiotoxicity [27]. It has been reported in 1% of patients, occurs shortly after drug administration, and is often associated with pre-existing cardiac disease [28]. Acute effects are usually clinically manageable [29].

It occurs when high doses of the drug are used and results in chest pain, electrocardiographic (ECG) abnormalities [6], mostly in ST-T [15], QT interval prolongation [21], pericarditis, or myocarditis. In a study focused on breast cancer, four of ten patients showed signs of cardiotoxicity before the end of DOX therapy as indicated by abnormalities in ECG [30].

However, studies suggest that the absence of symptoms of heart disease during or soon after the use of DOX does not preclude the appearance of cardiotoxicity many years later, known as chronic DIC [31]. Buzdar et al. examined 534 breast cancer patients treated with DOX. The development of congestive heart failure (CHF) caused by DOX therapy was found as early as 1 month after the end of treatment and affected 2% of the patients [30].

Early chronic cardiotoxicity occurs approximately within 1 year after completion of treatment. It manifests as dilated cardiomyopathy resulting in LV contractile dysfunction, ECG changes, arrhythmia, and left ventricular dysfunction (LVD) [32]. In a retrospective study conducted by van Hoff and co-workers comprising 4018 patients, DOX-induced CHF occurred in 88 (2.2%) of the patients evaluated between 0 and 231 days after the last DOX administration and manifested clinically as tachycardia, liver and myocardial enlargement, and pleural effusions [15].

Late cardiotoxicity has been observed in 8.3% of patients between 1 and 30 years post-treatment [33]. It usually leads to severe cardiomyopathy [34] and HF [27, 32, 34–37]. Important observations come from the follow-up of child cancer survivors treated with DOX. As many as 50% of them had abnormalities in echocardiography (ECHO) after DOX treatment [30]. A trial conducted by Lipshultz et al. involving cardiac evaluation, examined a group of 115 surviving children who were treated with mostly DOX for acute lymphoblastic leukemia. LV abnormalities were found on ECHO in 57% of patients, such as increased afterload, decreased contractility, reduced fractional shortening (FS), and LV wall thickness. One year after completion of DOX chemotherapy, 11 patients developed CHF. In two of these cases, a heart transplant was required [6].

In a retrospective study conducted by Steinherz et al. it was observed that 10 years after treatment with ANT about 23% of patients developed cardiac dysfunction, such as arrhythmia or HF [38]. Kumar et al. described a case of a 57-year-old woman treated with DOX at an age of 40 for breast cancer. Symptoms of HF in the form of progressive dyspnea, tachycardia, and hypertension appeared in this patient 17 years after the end of DOX treatment. ECHO examination showed significantly reduced LVEF (20%) and reduced contraction of LV. All of these symptoms have been classified as late cardiomyopathy associated with DOX administration [39].

The cardiotoxicity induced by DOX therapy is highly dose-dependent. Curigliano et al. found that the risk of HF is positively correlated with the cumulative DOX dose [40]. It increases by 3–5% after a dose of 400 mg/m^2^ and by 18–48% after a dose of 700 mg/m^2^ of DOX [46]. The incidences of cardiomyopathy are 5% at a cumulative dose of 400 mg/m^2^, 26% at 550 mg/m^2^, and 48% at 700 mg/m^2^ [40]. The incidence of cardiotoxicity may be underestimated due to the short follow-up of patients during and after completion of DOX therapy, as well as the use of less sensitive diagnostic methods [6] rather than two-dimensional or three-dimensional transthoratic ECHO, GLS, multigated acquisition scanning (MUGA), and cardiac magnetic resonance imaging (CMRI) [41].

Nevertheless, it is emphasized that there is no DOX dose that can be considered completely safe. The statement is based on a study of long-term pediatric acute lymphoblastic leukemia survivors. Patients were divided according to the dose of DOX they received: low dose < 300 mg/m^2^, moderate dose 300 to 400 mg/m^2^, and high dose > 400 mg/m^2^. ECHO showed that cardiac abnormalities such as reduced LV contractility and reduced LV mass and size affected patients in all DOX-treated groups (low-, moderate-, and high-dose groups). Even low cumulative DOX doses of less than 300 mg/m^2^ do not guarantee the absence of future cardiotoxic events [16, 42].

## Mechanism of DOX-induced cardiotoxicity

DOX has been a cornerstone in the treatment of various cancers due to potent antineoplastic properties. However, its clinical utility is significantly constrained by its cardiotoxic effects, which can lead to severe and sometimes irreversible heart damage. The cardiotoxicity of DOX is dose-dependent, thereby posing a considerable risk for patients undergoing chemotherapy. In order to develop effective protective strategies, it is therefore necessary to gain a deeper understanding of the underlying mechanisms.

The first of these mechanisms is oxidative stress and the associated production of reactive oxygen species (ROS) resulting from the redox reactions that DOX undergoes in cardiac cells. Consequently, substantial damage is incurred to cellular components, including lipids, proteins, and DNA. Oxidative damage leads to progressive impairment of cardiomyocyte function and viability, which contributes to the overall cardiotoxic effects of DOX.

The second mechanism is the inhibition of the enzyme topoisomerase II-beta, which is critical for DNA replication and repair. By interfering with the activity of this enzyme, DOX induces DNA damage and disrupts the normal cell cycle. In cardiac cells, this inhibition can result in cell death and contribute to the structural and functional damage.

**Fig. 1:**
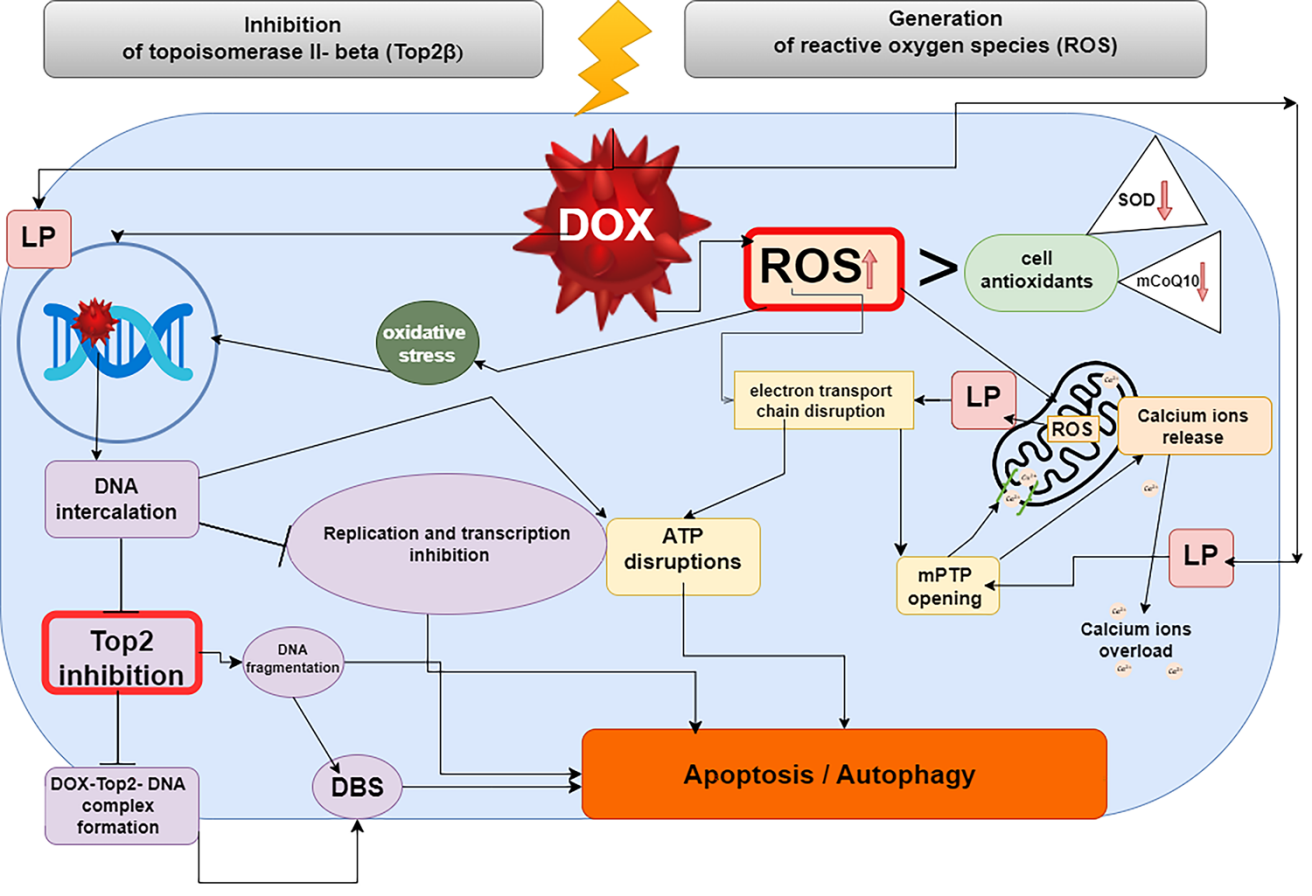
Two main mechanisms underscore DOX cardiotoxicity. ATP, adenosine triphosphate; DNA, deoxyribonucleic acid; DOX, doxorubicin; DSB, double-stranded DNA breaks; mCoQ10, mitochondrial coenzyme q10; mPTP, mitochondrial permeability transition pore; LO, lipid peroxidation; SOD, superoxide dismutase; ROS, reactive oxygen species; Top2β, topoisomerase IIβ

Collectively, these mechanisms lead to the characteristic cardiac dysfunction observed in patients receiving DOX chemotherapy. A comprehensive description of these two mechanisms is provided below (Fig. 1).

### Oxidative stress formation and generation of reactive oxygen species (ROS)

Oxidative stress is one of the main factors contributing to cardiovascular pathology. Its increased occurrence is observed especially in the case of cardiotoxicity induced by DOX administration in anti-cancer treatment. This phenomenon is defined as an imbalance in the cell between the production of antioxidants with ROS-detoxifying properties and the excessive production of ROS in noncancerous tissues, especially in the mitochondria of their cells. ROS under normal physiological conditions do not pose a danger to the cell, but during times of stress, such as chemotherapy, when produced in excess, they can destabilize cellular function and lead to irreversible damage [119].

ROS are formed when the quinone group, which is a component of the DOX structure, is reduced to a semiquinone radical via oxidoreductases, a class of enzymes that catalyze oxidation and reduction reactions, and reduced form of nicotinamide adenine dinucleotide (NADH). Subsequent oxidation of the semiquinone radical produces hydroperoxide (H_2_O_2_) and hydroxyl radical (•OH). This occurrence is commonly referred to as the “redox cycles” [43, 44]. Free radicals can easily cause damage to lipid membranes, proteins, and deoxyribonucleic acid (DNA) [45]. As a result of ROS-generating mechanisms, there is an imbalance between the action of ROS and the biological ability of the cell to repair ROS-induced damage and produce antioxidant enzymes. Since the heart lacks many antioxidant enzymes that convert ROS, the presence of DOX interferes with its activity [47].

DOX-related oxidative stress mediated by ROS in cardiomyocytes causes oxidation and degradation of biological membranes, i.e., lipid peroxidation. As a result of oxidation of unsaturated fatty acids that make up cell membranes, dangerous compounds are formed in the cell. Oxidative stress induced in cardiomyocytes by the formation of ROS causes an increase in the levels of lactate dehydrogenase (LDH), creatine phosphokinase (CPK), and malondialdehyde (MDA). LDH levels in the blood increase when the integrity of the cell membrane of cardiomyocytes is damaged due to lipid peroxidation, cell lysis, and the release of LDH into the bloodstream. An elevated level of CPK, a marker of free myoglobin indicating myocyte damage, occurs as a result of damage to cardiomyocytes with a high demand for adenosine triphosphate (ATP). The level of MDA, a highly toxic product of fatty acid peroxidation, increases due to its sensitivity to ROS and leads to unfavorable interactions with protein DNA in the cell [54]. The consequence is cardiomyocyte death through necrosis and activation of proapoptotic proteins providing apoptosis [48].

In a study of the effects of oxidative stress induced by the administration of DOX by an intraperitoneal injection (ip) at a dose of 20 mg/kg in male albino Wistar rats resulted in an increase in serum LDH and CPK compared to saline-treated controls. MDA levels in heart tissue were also elevated compared to those in healthy rats [55].

In a study by Wu et al. it was shown that free radicals acidified the cellular environment in cultured rat cardiac myoblasts and impaired the mechanistic function of the heart [46].

DOX-induced excessive production of ROS also causes an increase in nitric oxide (NO) in the cell, the concentration of which in the normal condition is initially low. NO formation occurs with the involvement of nitric oxide synthase (NOS), during the catalysis of L-arginine nitrogen oxidation reaction. There are three types of NOS: endothelial nitric oxide synthase (eNOS), neuronal nitric oxide synthase (nNOS), and inducible nitric oxide synthase (iNOS) [120]. Interaction with DOX results in overproduction of iNOS, especially mitochondrial-inducible nitric oxide synthase (i-mtNOS) which leads to excessive NO production in mitochondria [121]. The effect of further transformation is the formation of peroxynitrite (ONOO-), which is highly toxic to cells and leads to disruption of cellular homeostasis at multiple levels [122].

On the other hand in the presence of DOX, eNOS molecules are disconnected, leading to the overproduction of dangerous superoxide anions [123].

Superoxide anions can be removed by an enzyme called superoxide dismutase (SOD). It has been shown, however, that DOX supply causes a decrease in the activity of this antioxidant [33]. In a study conducted by Sankaran Rajagopalan et al. supply of SOD (600 units/mL) to DOX-perfused rat hearts using the Langendorff method caused inhibition of superoxide anions and H_2_O_2_ production [124].

ROS have a particular predisposition to attack mitochondria abundantly located in cardiomyocytes. By accumulating on the inner side of the mitochondrial membrane, through the action of a selective protein such as cardiolipin, ROS disrupts mitochondrial respiration by inhibiting the expression of electron transport chain proteins. This causes a decrease in the mitochondrial membrane potential, opening of the mitochondrial permeability transition pore (mPTP), passage of ROS molecules into the mitochondrial interior, damage to mitochondrial DNA, and release of calcium ions into the cell, leading to calcium overload [125].

Since ROS interfere with mitochondrial respiration by inhibiting the expression of electron transport chain proteins, there is also disruption of the non-protein electron carrier molecule Q10, the so-called mitochondrial coenzyme q10 (mCoQ10), a potent antioxidant. mCoQ10 conditions proper ATP synthesis and maintenance of mitochondrial membrane potential. On the other hand, protein complexes of respiratory chain electrons (I and III) bound to CoQ10 provide a site for the formation of mitochondrial reactive oxygen species (mRFTs) [126].

In a study conducted on the hearts of Wistar rats, taken on day 16 of the experiment from animals given DOX and DOX + CoQ10 intravitally, it was observed that in the group of animals given CoQ10, there was the following: decreased expression of BAX, known as a regulator of proapoptosis, and increased expression of Bcl-2, known as an anti-apoptotic regulator. A difference was also observed in anti-caspase-3 antibody staining, which identified a more extensive cytoplasmic response to anti-caspase-3 in the LV heart tissue of rats receiving DOX alone compared to the group receiving DOX + CoQ10. *Similar results were obtained using iNOS antibody* staining [127].

Another pathway of ROS formation is through iron oxidation–reduction cycles and accumulation of DOX-iron complexes in the mitochondria [44].

Oxidative stress in cardiomyocytes can be induced in response to DOX through activation of the renin–angiotensin–aldosterone system (RAAS) system, which initially maintains cardiac output (CO), but ultimately its continued action is classified as adverse in DIC [53].

### Inhibition of topoisomerase II-beta (Top2β)

Topoisomerase (Top) is an enzyme responsible for maintenance of DNA conformation, involved in processes such as replication and transcription. This is why Top has become a target for anti-cancer drugs such as DOX not only in onco-cells, but also in physiologically healthy cells [56]. There are currently two types of Tops:

Tops class 1 and Tops class 2: Tops1 untangles the double helix of DNA using single cuts, cutting only one of the two DNA strands (single-strand breaks (SSB)), while Tops2 cuts two strands simultaneously (double-strand breaks (DSB)) [128].

Among the Top 2 class, α and β isoforms are distinguished. A strong expression of Top2 β has been identified in all cells, and an increased expression of Top2α in cells that divide strongly during phases of the cell cycle [129].

In cardiomyocytes, there is a form of Top called Top2β expressed [56]. Despite the effective anti-cancer strategy associated with DOX, a number of irreversible reactions occur in healthy cardiomyocytes, resulting in these cells entering the death pathway. The mechanisms of DOX’s impact on Top2β and their consequences are presented below.

DOX belongs to one of two classes of anti-cancer drugs that actively target Top2. A class of these drugs is called “Top2 poisons,” while a second class of drugs belongs to the so-called Top2 catalytic inhibitors. The main difference in the action of these two classes of drugs is the formation of covalent bonds between the enzyme and DNA. While DOX is capable of forming covalent complexes, class 2 drugs are not characterized by this property, but by their ability to catalytically inhibit Top2 without forming Top2-DNA complexes [130].

The main function of Top2β is to simultaneously cut the DNA double strand by binding the 5′-phosphodoxyribosyl end to subunits of the enzyme. When Top2β is bound to DNA helix in order to cause controlled DSBs, catalytic activity of the enzyme is inhibited as a result of blocking its rotation, and DOX attaches and forms a ternary Top2β-DNA-DOX complex. Due to the presence of a sugar residue in the DOX structure, the stability of the ternary complex is ensured. As a result, the rejoining of DNA strands is inhibited, fragmentation of DNA in the cell nucleus takes place, and ATP synthesis in the mitochondria is disrupted. All this leads to cell death [56].

Additionally, DOX intercalates between DNA base pairs in place of the sequences 5′-GC-3′, 5′-CG-3′. Formation of covalent bonds (N-C-N) with the 2-N guanine amino group between the DOX aromatic ring and base pairs with the DNA amino sugar happens. The formed adduct is stabilized by hydrogen bonds and van der Waals forces [131].

It results in elongation of the DNA helix, loss of flexibility, and distortion of the double strand resulting in inhibition of replication. As a consequence, mitotic catastrophe occurs [57–59].

### The various other mechanisms of DOX-induced cardiotoxicity

#### Loss of mitochondrial cell membrane integrity

Another consequence of DOX influx in the cells is loss of mitochondrial cell membrane integrity (Table 1). DOX induces the release of mitochondrial cytochrome c oxidase (COX), a protein located in the inner mitochondrial membrane, involved in electron transport through the respiratory chain. The oxidation of cytochrome c by DOX-induced ROS results in its oxidation, release of apoptosis-inducing factor (AIF) into the cytoplasm, the formation of the apoptosomes, caspases activation, and disruption of gene expression [36]. Thus begins the initiation of the process of the internal cell apoptosis pathway.

**Table 1:** The various other mechanisms of DOX-induced cardiotoxicity

Mechanism	Results	References
Loss of mitochondrial cell membrane integrity	Reaction with the protein COX, disruption of the membrane potential, impaired ATP production, impaired cardiomyocyte contractility formation of caspase-activating proteins complex, autophagy and apoptosis activation	[78]
Dysregulation of calcium regulatory proteins	Activation of mPTP, membrane potential loss, release COX, disruption of mitochondrial ATP biogenesis and antioxidant functions, autophagy and apoptosis activation	[79]
Accumulation of iron ions	Inhibition of ferritin, formation of DOX-complexes with free iron and metabolites: DOXol, IRP1, and IRP2, autophagy and apoptosis activation	[64]
Fibrosis initiation	Increase of TGF-β1, occurrence of hypertrophy, apoptosis activation	[73]
AMPK inhibition	Decrease in ACC activity, PKB and MAP activation	[54]
Autophagy activation	Activation of Bcl-1, AGT, formulation of autophagosome, AMPK and mTOR kinases inhibition, increase in p53 protein level	[36]
Apoptosis activation	Activation of BAX proapoptotic proteins, caspases and p62 protein, disturbances in PI3K/AKT pathway, degeneration of cell organelles, cardiomyocyte death and replacing it by fibroblasts collagen production	[36, 73]

*ACC* acetyl-CoA carboxylase, *AMKA* 5'AMP-activated protein kinase pathway, *ATP* adenosine triphosphate, *BAX* Bcl-2-associated X protein, Bcl-1 beclin-1, *COX* cytochrome c oxidase, *mPTP* mitochondrial permeability transition pore, *DOXol* doxorubicinol, *IPR1* iron regulatory proteins 1, *IPR2* iron regulatory proteins 2, *MAP* mitogen-activated protein kinase, *PI3K/AKT* phosphatidylinositol-3-kinase/protein kinase B survival pathway, *PKB* protein kinase B, *mTOR* mechanistic/mammalian target of rapamycin, *TGF-β1* transforming growth factor β1

**Table 2:** The mechanisms of cardioprotective action of the apelinergic system in DOX-induced cardiotoxicity in preclinical research

*Subject of research*	*Cardiovascular model injury results*	*Apelinergic system activation*	*Apelinergic system cardioprotective action*	*References*
In vivostudies				
APL (APL-/-) male and female deficient mice APJ (APJ-/-) male and female deficient mice Both under stress conditions	↓LV systolic function ↓Cardiac minute volume	-	↑EF ↑FS ↓LVESD	[95]
APL-KO male and female mice	↓Cardiac contractile effect	APL-13 infusion at a dose 1 mg/kg per 24 h via mini-osmotic pumps	↑FS ↓LVESD	[96]
Male Wistar rat model of ISO myocardial injury	↓LVESP ↑LVEDP ↑Oxidative stress	APL at a dose 5 and 20 nmol/kg/day for 5 days	↑LV + dp/dt / LV -dp/dt ↓BNP ↓MDA ↓LDH	[18]
Male Wistar rat model of MI by LAD	↑Oxidative stress	ip administration of [Pyr1]-APL-13 at a dose 10 mol/kg/ for 5 days	↓MDA, ↓LDH, ↓CK-MB	[97]
Male Wistar rat model of I/R by LAD	↑ Myocardial infarct size ↑Oxidative stress	Bolus intravenous injection of APL-12 at a dose of 0.35 μmol/kg	↓ IS/AR ↓CK-MB ↓LDH	[98]
Male Wistar rat model of I/R	↑Oxidative stress ↑Preload ↓Apoptosis	APL-13 perfusion at a dose of 30 pmol/L	↓Coronary perfusion pressure ↓LVEDP ↓LDH ↓MDA	[99]
Female Lewis rats model of MI	↓Cardiac contractile effect	iv infusion of PEG-APL-36 30 nM and APL-36 30 nM, at a dose of 1 mL/20 min/250 g for 20 min	↑EF	[104]
SD rat model of acute MI (unspecified gender)	↓Heart hemodynamics	iv administration of APL-13 at dose of 10 μg/kg per minute	↑CO ↑SV	[20]
Male rat model of right ventricular hypoxia	↓Myocardial contractile force	APL-12 pretreatment at a dose of 10 ~ 70 nM	↑[Ca2 +]I transient	[106]
Male SD rat model of LAD-induced MI	↑Fibrosis ↓Contractile effect ↑Oxidative stress	ip administration of APL-13 at a dose 10 nmol/kg/day for 28 days	↓Collagen I ↓Collagen II ↓TGF-β ↑EF ↑FS	[107]
C57BL6/J male mouse model of pressure overload	↑Fibrotic processes ↑Inflammation	APL administration at doses 1, 10, 100 nM	↓a-SMA ↓Collagen ↓TGF-β ↓IL-6	[108]
Male Wistar rat model of HF	↑Apoptosis	ip administration of [Pyr^1^]APL-13 at a dose of 10 nmol/kg/day for 5 days	↑Bcl-2 ↓BAX ↓Caspase-3	[109]

*Subject of research*	*Results*	*Apelinergic system activation*	*Apelinergic system cardioprotective action*	*References*
In vitro studies				
Isolated LV cardiomyocytes of female APL and APJ-KO mice	↓Systolic function ↓Systolic and diastolic times	-	↑EF ↑FS ↑CO	[95]
Male Wistar rat neonatal cardiomyocytes of subjected to H/R	↑mRNA expression of APL and APJ	APL-13 perfusion at a dose of 30 pmol/L	↓I/R	[99]
Wistar rat cardiac neonatal myocyte cultures (unspecified gender)	-	APL therapy at a dose of 100 nM	↓H_2_O_2_ production	[101]
Isolated male SD rat cardiomyocytes	-	APL-16 at a dose of 1 nM administration	↑([Ca2 +]i) transients ↑FS ↑SERCA activity	[105]

*a-SMA* smooth muscle a-actin, *APL* apelin, *APJ* apelin receptor, *BAX* Bcl-2-associated X protein, *Bcl-2* B-cell lymphoma 2, *BNP* B-type natriuretic peptide, *Ca2 +* calcium ion, *CK-MB* creatine kinase-myoglobin binding, *CO* cardiac output, *EF* ejection fraction, *FS* fractional shortening, *I/R* ischemia/reperfusion injury,* H*_*2*_*O*_*2*_ hydroperoxide, *IL-6* interleukin-6, *IS/AR* myocardial infarct size expressed as percentage of the area at risk, *ISO* isoproterenol, *LAD* left anterior descending artery, *LDH* lactate dehydrogenase, *LV* left ventricle, *LV* + *dp/dt / LV -dp/dt* left ventricle positive/negative pressure, *LVESD* left ventricular end-systolic diameter, *LVEDP* left ventricular end-diastolic pressure, *LVESP* left ventricular end-systolic pressure, *KO* knockout, *MDA* malondialdehyde, *MI* myocardial infarction, *[Pyr1]-APL-13* pyroglutamyl form of apelin-13, *SD* Sprague–Dawley rat, *SERCA* sarcoplasmic/endoplasmic reticulum Ca2 + -ATPase, *TGF-β* transforming growth factor-β

Impaired mitochondrial function can result in impaired ATP production in cells and impaired cardiomyocyte contractility [60].

Tanaka et al. [61] treating HCF with DOX (0.1 μM) using the MitoTracker Red method to assess mitochondrial activity, showed that DOX decreases mitochondrial membrane potential. Exposure to DOX also induced the initiation of mitophagy (confirmed by staining with mitophagy dye), that is, selective digestion of mitochondria. This corresponded to increased expression of biomarkers occurring in the process of autophagy cell death: phospho-PI3 kinase class III (p-PI3KC3), p62, and LC3 [61].

In a prospective study of 100 patients with solid or hematologic malignancies receiving DOX chemotherapy (53% of patients), ECHO was performed to assess the effect of DOX on LV systolic function. The baseline ECHO and subsequent measurements up to the last one, 1 year after the end of therapy, showed a decrease in parameters reflecting LV systolic function. The EF (65.9 ± 0.6% vs. 61.6 ± 0.7%) and FS (39.7 ± 0.5% vs. 36.5 ± 0.6%) decline parameters progressed during and even after therapy [62].

#### Dysregulation of calcium regulatory proteins

Different factors leading to DOX cardiotoxicity include dysregulation of calcium regulatory proteins. DOX interferes with calcium metabolism. As a result, the ability of mitochondria to store calcium is impaired. Doxorubicinol (DOXol)—a hydroxyl metabolite of DOX, by interacting with voltage-dependent channels: sarcoplasmic/endoplasmic reticulum Ca^2+−^ATPase (SERCA), ryanodine receptor Ca^2+^-release channel (RYR), and Na^+^-Ca^2+^ exchanger (NCX)—disrupts the homeostasis of calcium ion flow into and out of the cell. In addition, it initiates the release of calcium ions from the endoplasmic reticulum [63, 64].

In a study conducted by Pecoraro et al. on cardiomyocytes from DOX-treated mouse hearts, DOX was found to increase the expression of connexin 43 (Cx43) [65]. Cx43 is a protein belonging to the gap junction family responsible for protecting against calcium ions overload by forming hemichannels that promote ion transport between cells and protect against impaired mitochondrial permeability caused by DOX [66]. Thus, the presence of DOX contributes to the release of calcium from mitochondria, as evidenced by the high expression of Cx43 [65]. However, Wu et al. in an ex vivo study on Langendorff guinea pig male hearts using optical mapping, showed that DOX administration decreased intracellular calcium levels, which would correlate positively with a decrease in cardiomyocyte contractile function [67]. The overload of calcium is the main reason for membrane potential loss. Due to this action, the cell membrane ruptures, and COX is released and activates caspases. Thus, it contributes to the cell apoptosis pathway [63].

#### Accumulation of iron ions

Additionally, DOX affects the distribution of iron ions in cells and ultimately leads to ferroptosis by lipid peroxidation death [68]. Upregulation of the transferrin receptor (TfR) and inhibition of ferritin are the underlying causes of the accumulation of iron inside cells, especially in mitochondria. Through the formation of DOX complexes with free iron and metabolite DOXol, ROS are formed [69, 70]. Inactivation of iron regulatory proteins 1 and 2 (IRP1 and IRP2) results in the modification of gene expression responsible for iron balance [71]. In a study conducted on small cell lung cancer (GLC4) and myeloblastic leukemia (K562) cell lines by Brazzolotto et al. [72] it was observed that DOX exposure causes reversible inactivation of IRP1, a master regulator of cellular iron homeostasis. In contrast, IRP1 inactivation was not detected in the sublines of the aforementioned DOX-resistant cancers. This provides evidence of dysregulation of cellular iron levels by DOX [72].

#### Fibrosis initiation

The action of DOX also causes fibrosis. It occurs as a result of excessive oxidative, inflammatory reaction, and resulting cell death. An in vitro study on human cardiac fibroblasts (HCFs) noted that DOX supply results in increased mRNA expression of inflammatory cytokines after such as interleukin-1, interleukin-1 B, and toll-like receptor 9 acting as a warning to the immune system. Also, the levels of actin alpha 2, smooth muscle, galectin 3, and tissue inhibitor of metalloproteinases 1 were higher in HCFs receiving DOX compared to the control group [61]. The apoptotic cardiomyocytes are replaced by collagen production by fibroblasts (Table 1). The main marker of fibrosis and cardiomyocyte hypertrophy in DIC is transforming growth factor β1 (TGF-β1). In a rat model of chronic DIC due to DOX administration for 4 weeks, TGF-β1 was assessed in rat myocytes by immunohistochemical staining. A significant increase in TGF-β1 was observed in DOX-treated rats compared to the control group. In addition, Masson’s trichrome staining of cardiac tissue showed the greatest increase in collagen volume fraction and perivascular collagen area in the DOX group versus healthy animals (Table 1).

#### Inhibition 5'AMP-activated protein kinase pathway (AMPK)

DOX inhibits the protein complex AMPK, an enzyme active to low levels of ATP, that initiates the uptake and oxidation of glucose and fatty acids. It leads to decreased activity of the acetyl-CoA carboxylase (ACC), regulator of the rate of fatty acid oxidation. It results in protein kinase B (PKB) and mitogen-activated protein kinase (MAP) involved in directing cellular responses to kinase activation. Both are involved in regulating the processes of proliferation, cell survival, and apoptosis (Table 1).

#### Autophagy activation

DOX can initiate autophagy, a process taking part in cellular homeostasis, which not only can occur under normal conditions but is also triggered under stressful states, such as lipid peroxidation of cells causing DIC. Its dysregulation is severe and leads to excessive cell death [74]. DOX inhibits the mechanistic/mammalian target of rapamycin (mTOR)—kinases promoting the PI3K/AKT (Table 1).

These factors lead to the formulation of autophagosome from the endoplasmic reticulum resulting in the formation of a ternary complex consisting of Unc-51-like kinase 1(ULK1), RB1-inducible coiled-coil protein 1 (FIP200), and autophagy-related gene (Atg) [36]. It further induces recruitment of beclin-1 (Bcl-1), Atg proteins, and activation of p62 known as a driver of the apoptosis process [75]. Cao et al. [76] infused neonatal rat ventricular myocytes (NRVMs) with DOX. The 24-h infusion showed increased expression of LC3-phosphatidylethanolamine conjugate (LC3-II), a standard marker for autophagosomes, caspase-3, Bcl-1, and p62, a marker of autophagy flux [76].

In a study conducted by Wang et al. on cell cultures of the H9c2, a subclone of the original clonal cell line derived from rat embryonic heart tissue was subjected with DOX (1 μm). It has skeletal muscle traits, making it a uniquely suitable model for studying cardiovascular pathology. In this cell line, DOX administration inhibits the phosphorylation of AMPK and increases the levels of p53 known as a protein-regulated apoptosis and tumor suppressor [77].

#### Apoptosis activation

DOX activates apoptosis in two ways: the intrinsic and the extrinsic pathways.

The intrinsic upregulation of p53 protein is associated with the release of proapoptotic factors, including Bcl-2-associated X protein (BAX) proteins of the B-cell lymphoma 2 (Bcl-2) family. The action of BAX proteins is to increase the permeability of the pores in the outer membrane of mitochondria. This results in loss of membrane potential, calcium ion entry into the cytoplasm, and calcium overload [36]. The phosphatidylinositol-3-kinase/protein kinase B survival pathway (PI3K/AKT) has been identified as the most sensitive to proapoptotic signals in the cell [49].

The extrinsic pathway of apoptosis connected with DIC is associated with upregulation of death receptors named death receptor 4 (DR4) and death receptor 5 (DR5), Fas receptor, and tumor necrosis factor receptor 1 (TNFR1) [50]. It contributes to the activation of pro-inflammatory nuclear factor of activated T cells (NFAT) and nuclear factor-κB (NF-κΒ). Caspases—proteins belonging to the cysteine-aspartic acid protease family involved in the execution phase of cell apoptosis—are then activated and cell death occurs [36]. In a study by Ueno et al. [51] on primary cultured cardiomyocytes prepared from hearts of neonatal Sprague-Dawley (SD) rats treated with DOX, a significant increase in caspase-3 activity was observed compared to the untreated group. To estimate apoptosis, terminal deoxynucleotidyl transferase dUTP nick end labeling (TUNEL) staining was performed on the myocardium of 8-week-old SD rats. TUNEL staining in the ventricles treated with DOX was significantly higher than in the saline group. This indicates the apoptotic properties of DOX [51].

In a study by Tao et al. [52] activation of apoptotic pathways with increased activity of MAP kinases and p38, p53 proapoptotic transcription factors was observed in cardiomyocytes administered DOX in a rat model with inducible ischemia and reperfusion injury (I/R). Also caspase-3 was identified [52].

As can be seen from the abovementioned mechanisms of action of DOX, its use carries many risks for homeostasis and cellular function in the body. Therefore, new therapeutic targets are being sought to prevent or improve the cardiovascular parameters like EF, FS, and CO disturbed by DOX action in particular, and to maintain these parameters for a long time. Among them, due to its unique properties and effects on the cardiovascular system, may be the apelinergic system with components APL, ELA, and APJ.

## Apelinergic system

APL is a selective endogenous ligand discovered from bovine stomach extracts [85]. This active peptide belongs to the adipokine family and is highly connected with G-protein-coupled receptors (GPCRs) which is widely implicated in the control of cardiac function. APL has several analogs with amino acid lengths of 12, 13, 17, and 36, each formed by C-terminal cleavage of a 77-amino acid precursor of preproapelin encoded on X chromosome q25-26 [80–84, 86, 87].

APL was detected in early embryonic development during cardiovascular formation. Every form of APL has been found in the plasma and cardiovascular system in rodents and humans [88]. APL-13 is considered the most biologically active [86]. APL has been localized in the central nervous system [88]. APL levels correlate with the amount of vasculature in organs [89].

Kawamata et al. [90] observed that higher concentrations of APL were present in the lungs, cardiovascular system, and spleen, and are proportional to the level of their vascularization. Factors such as insulin, growth hormone, and tumor necrosis factor α (TNF-α) stimulate the synthesis of this adipokine, while cortisol acts to limit production [90].

The apelinergic system also consists of another ligand that acts with APJ [91]. In 2013, ELA/Toddler was discovered by the Reversade group (2013) studying signals that regulate early development in zebrafish embryos. It has confirmed the existence of an earlier ligand for apelin receptor. ELA/Toddler is classified as an apelinergic system component encoded by the APELA gene located on chromosome 4. Main ELA consists of 32 amino acids; however, there are also shorter forms that possess the same activity ELA-22 or ELA-11. ELA amino acid sequence is at 25% similar to the APL; and therefore, it seems that ELA can activate the APJ and exert an APL-like effect [92, 93].

**Fig. 2:**
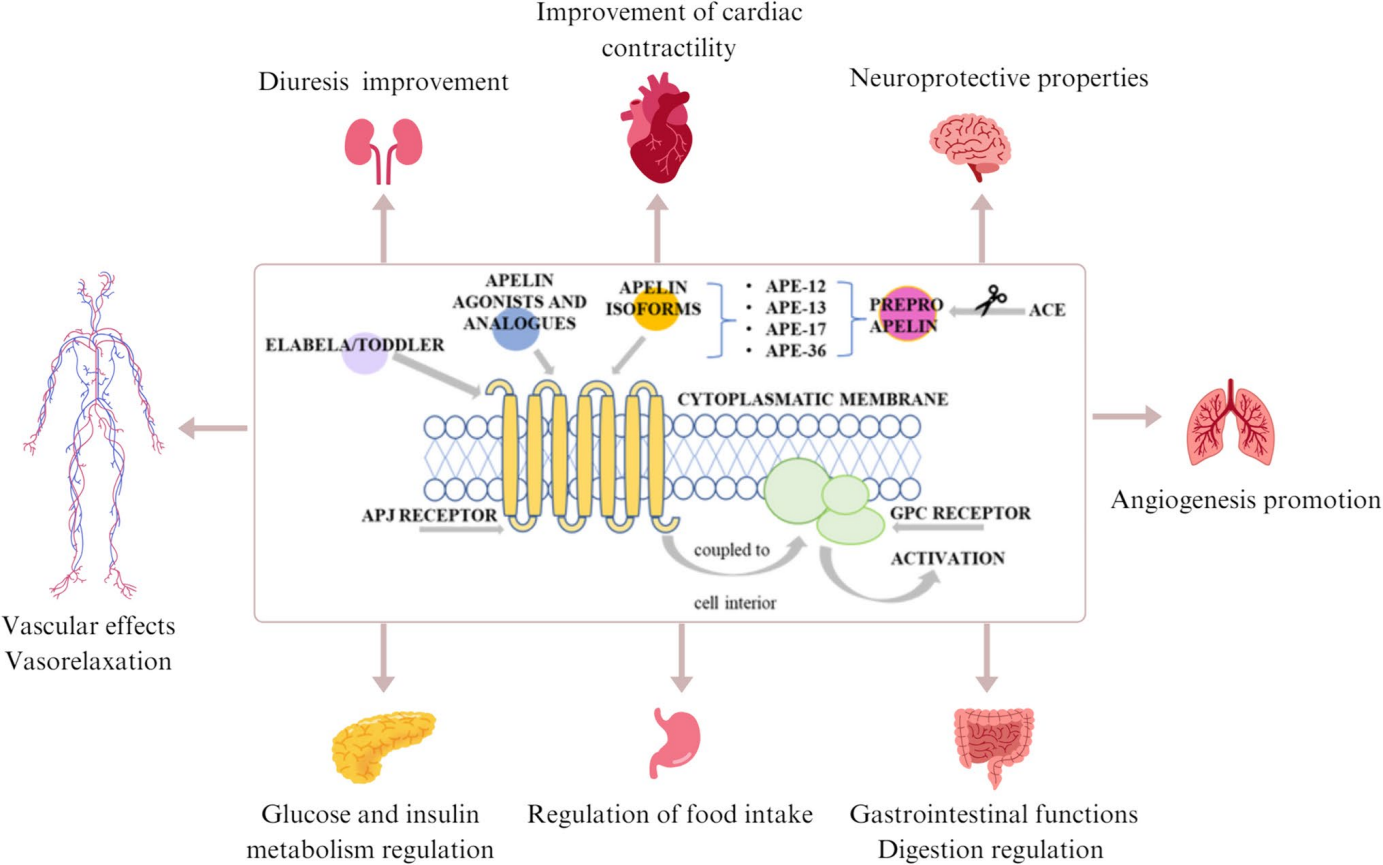
Apelinergic system structure and function. ACE, angiotensin-converting enzyme; APE-12, -13, -17, -36, apelin 12, 13, 17, 36; APJ, apelin receptor; GPC receptor G-protein-coupled receptor

APJ was discovered in1993 by O’Dowed and identified as a putative receptor, a member of the G protein-coupled receptor superfamily. The APJ demonstrates sequence 40–50% homology with the Ang II receptor AT-R1, but Ang II does not activate and seems to act as a counter-regulator. APJ is present in the heart, blood vessels, brain, lungs, kidneys, and adipose tissue [94]. Attachment of APL ligand to the APJ and interaction of the receptor with G proteins trigger the essential signaling pathways of PI3K/AKT, protein kinase C (PKC), and extracellular signal-regulated kinase 1/2 (ERK 1/2). These pathways are responsible for regulating oxidative stress, proliferation, and cell survival pathways [85] (Fig. 2).

## The apelinergic system in cardiovascular diseases (preclinical studies)

Both in vitro studies on isolated cardiomyocytes and in vivo studies on mouse and rat models provide valuable information on the beneficial effect of APL and its receptor in cardiovascular pathologies.

In vivo studies were conducted on APL (APL-/-) and APJ (APJ-/-) deficient mice. Animals were maintained under normal and stressful conditions (exercise test). ECHO indicated impaired cardiac function (reduced fractional shortening) in both APL and APJ knockout (KO) mice.

APL reduced end-systolic pressure under normal and stress conditions. Lack of function of the APJ/APJ signaling pathway contributed to reduced cardiac minute volume in stress response. Observations of the offspring of APJ knockout (APJ-KO) mice provided information on the incidence of cardiovascular malformations such as significantly reduced FS and EF, while the APL KO mice were born healthy and developed normally. Through the use of stimulation of isolated LV cardiomyocytes of female mice, it was revealed that in the APL and APJ-KO groups, the cardiomyocytes were characterized by reduced systolic function and slower systolic and diastolic times. This demonstrates that the mechanism of action of the APJ and APL affects cardiac function, and its absence promotes deterioration of cardiac parameters such as EF, FS, and CO [95].

Similar results were obtained by Kuba et al. [96] who investigated the effect of APL on cardiac contractile disturbance and associated LVD on APL gene-deficient male mice. Mean arterial blood pressure (MAP) in anesthetized 3-month-old APL-KO and wild-type (WT) male mice was compared. APL-deficient mice were shown to have no dysfunction associated with LV hypertrophy, and body weight to heart weight ratio was within normal limits. ECHO measurements, however, indicated a significantly progressive reduction in FS in 6-month-old APL-KO males. This correlated positively with an increased left ventricular end-systolic diameter (LVESD). APL-KO males were subjected to a continuous, 2-week infusion of APL-13 implantation of mini-osmotic pumps. APL-13 supplementation reversed the decreasing FS, while this effect was not observed in WT mice [96].

APL has shown protective effects in treating damaged myocardium. This was demonstrated by Jia et al. [18] in a model of isoproterenol (ISO) myocardial injury. The ISO-treated Wistar rat showed 49.7% lower positive LV pressure over time (dp/dtmax) and 53.1% lower negative LV pressure (dp/dtmax) values. Left ventricular end-systolic pressure (LVESP) was 23% lower, while left ventricular end-diastolic pressure (LVEDP) was 346% higher compared to the control group. ISO rats receive ip APL at a dose (5 and 20 nmol/kg/day, respectively) for 5 days. As a result, there was an increase in the above parameters by 51%, 53%, and 23%, respectively. LVEDP decreased by 36%. The mRNA expression levels of B-type natriuretic peptide (BNP), MDA, and LDH were increased in ISO-treated animals compared to the healthy group while APL treatment reduced this effect [18].

In a male Wistar rat model of MI, a highly potent pyroglutamyl form of APL-13 [Pyr^1^]-APL-13 (10 mol/kg/day) ip was administered for 5 days after left anterior descending artery (LAD) banding. Serum samples obtained from blood collected on days 1, 3, 5, and 7 after MI were analyzed. The study revealed that APL is capable of lowering MDA, LDH, and CK-MB in serum [97]. It was also confirmed that the use of APL in a model of MI reduces its size. In an animal model (Wistar rat) of I/R by LAD banding, bolus intravenous injection of APL-12 at a dose of 0.35 μmol/kg reduces the ratio of myocardial infarct size (IS/AR) to 40% compared to the control group. Plasma showed that APL supply statistically significantly reduced CK-MB activity. LDH levels were reduced by 47% compared to the saline-treated control group. Similar observations were obtained by studying the structural analogs of APL-12 [MeArg1, NLe10]-A12 (I) and [d-Ala12]-A12 (II) in the same experiment [98]. In conclusion, these results suggest that APL can reduce the extent of myocardial damages in an animal model of MI.

APL has been proven to protect the heart from I/R injury by decreasing ROS and LDH level. It was observed a significant increase in mRNA expression of APL and APJ in cardiomyocytes of neonatal rats subjected to hypoxia/reoxygenation (H/R) injury and perfused hearts of male Wistar rats under I/R. Administration of APL-13 at a dose of 30 pmol/L for 30 min reduced the size of I/R. ECHO parameters, after APL-13 treatment in I/R injury, were close to those in the control group. There was a decrease in coronary perfusion pressure and LVEDP. It has been sighted that under the influence of APL-13 supply, inhibition of LDH leakage and MDA production took place. Reduction of apoptosis occurred due to downsizing of ROS production [99]. These results are favorable, especially in terms of APL’s reduction of oxidative stress parameters induced by ROS causing damage to cardiomyocytes and leakage of LDH and MDA, and reduction of CK-MB accompanied by DOX-induced cardiotoxicity.

In addition to the release of ROS by DOX, myocardial remodeling often occurs as a result of its action. It is indicated that APL can prevent myocardial hypertrophy. APL, in a dose-dependent manner via catalase, an antioxidant enzyme present in mitochondria and peroxisomes, prevents LV hypertrophy and reduces oxidative stress by inhibiting ROS production, such that it breaks down H_2_O_2_ to water and oxygen [100]. It was confirmed in a study conducted by Foussal et al. [101] on rat cardiac neonatal myocyte cultures. APL therapy at a dose of 100 nM reduced the growth of neonatal cardiomyocytes induced by serotonin (5-HT) at a dose of 10 µM, which activated intracellular signaling pathways involved in H_2_O_2_ production. APL blocked H_2_O_2_ production in cardiomyocytes, while cardiomyocytes treated with 5-HT alone showed its overproduction [101]. This confirmed the hypothesis that pretreatment with APL inhibits ROS production in vitro. Examining H9c2 cells exposed to DOX (1 µM) and incubated for 24 h, Yoon et al. [102] show that DOX leads to upregulation of hypertrophic markers, as manifested by increased levels of atrial natriuretic peptide (ANP), BNP, beta-myosin heavy chain (β-MHC), and myosin light chain-2 (MLC2). It was also noted that DOX induced an enhanced calcineurin/nuclear factor of activated T (CaN/NFAT) cells signaling pathway, which activates a pro-hypertrophic gene transcription program [102].

A study by Scimia et al. [103] identified a dualism of APJ action. Its effects on myocardial hypertrophy were highlighted. APJ-KO mice were subjected to sustained pressure overload by transverse aortic constriction (TAC). These animals exhibited a tendency to reduce myocardial hypertrophy in the future predisposing to HF. Thus, it was concluded that the mechanical response of APJ induces myocardial hypertrophy by recruiting β-arrestin—a protein that contributes to many aspects of GPCR signaling. Deletion of the gene encoding APL in APL-KO mice did not cause this effect. Comparison of measurements of sarcomere strength of cardiomyocytes isolated from WT+APL groups of mice with the APJ-KO+APL group provided information on the modulation by APL of the stretch response mediated by the APJ. This highlighted the possibility that inhibition/modulation of the APJ stretch response could be used to avoid pathological phenomena in the heart [103].

APL/APJ axis exerts a positive inotropic effect associated with an increase in the strength of cardiomyocytes contraction on. It is especially important in heart diseases associated with impaired EF and CO stroke volume (SV). Attenuation of these parameters commonly occurs in both early and late DIC and is an indicator of LV systolic dysfunction, especially EF [62].

PEGylated with polyethylene glycol APL-13 (PEG-APL-36) and APL-36 were administered intravenously (iv) to female Lewis rats in a model of MI. Cardiac function was monitored by ECHO. EF measurements in healthy rats showed comparable increases in this parameter with both types of APL. In the MI model, PEG-APL-36 prolonged and made stronger the inotropic effects in MI EF which were 30% for the APL-36 group and 40% for the PEG-APL-36 group, and for the control group, respectively 18% and 21% [104].

During a study on the cardioprotective effects of APL on acute MI and adverse myocardial remodeling, an increase in CO and SV was noted after APL supplementation. Examination of the parameters of CO, SV, and maximum ventricular rate in normal SD rats subjected to acute iv injection of APL-13 showed a dose-dependent increase in the values of these parameters. Administration of a dose of 10 μg/kg per minute resulted in the greatest increases in CO (15.4% from baseline) and SV (12.6% from baseline). No increase in HR was observed [20]. This result is consistent with a study on dogs with advanced HF [19]. It was noted that a similar response is obtained after providing BMS-986224, a potential APJ agonist, which caused an increase in CO (10–15%) [20].

DOX in mitochondria disrupts ion channels associated with the transport of calcium, which is required for contractile protein contraction when ATP-mediated myosin heads connect to actin. It has been demonstrated that APL is involved in the management of calcium ions. APL-16 loading of isolated SD rat cardiomyocytes resulted in increased intracellular calcium concentration ([Ca2+]i) transients, FS and SERCA. In the presence of chelerythrine chloride, a cell-permeable protein kinase C inhibitor, the inotropic effect of APL-16 ceased. It suggests a SERCA-dependent inotropic role for this peptide [105]. A similar result was obtained by Dai et al. finding that APL affects myocardial contractile force by increasing [Ca2 +]i levels. In an in vivo study on rats subjected to right ventricular hypoxia for 14 to 16 weeks, APL-12 injected into pre-hypoxic hearts at a dose of 10 ~ 70 nM directly increases the contractility of failing muscles compared to the healthy group [106].

DIC often results in the replacement of apoptotic cardiomyocytes by collagen and increase in marker of fibrosis: TGF-β1 [73, 117]. Zhong et al. [107] found that APL-13 alleviated cardiac fibrosis. Intraperitoneally supplementation of APL-13 (10 nmol/kg/day, for 28 days) in an animal model of LAD-induced MI (SD rats) alleviated fibrosis in the heart and improved ECHO parameters responsible for LV function. Protein levels in Western blot analysis of collagen I, collagen III, and transforming growth factor-b (TGF-β) were shown to be reduced in the myocardium of APL-13 treatment rats compared to the control group. Also in isolated cardiac fibroblasts (CF), the levels of oxidative stress and the fibrosis indicator TGF-β were reduced. Moreover in the same study it was revealed that levels of phosphoinositide 3-kinase (PI3K) and phosphorylation of Akt (p-Akt) increased in the presence of Ang II in CF. APL-13 blocked this increase, and increased EF and FS in rats with MI [107].

In turn, Pchejetski et al. [108] confirmed that APL reduces fibrotic processes in cardiac cells through TGF-β-dependent sphingosine kinase activation. Mouse CF prepared in a pressure-overload hearts model and from healthy hearts were studied. Dose-dependent administration of APL (1, 10, and 100 nM) was demonstrated to reduce fibroblast loading. The production of TGF-β, smooth muscle a-actin (a-SMA), and collagen was significantly inhibited at an APL dose of 10 nM. The findings were confirmed in vivo in C57BL6/J mice in a pressure overload model. Mice treated with APL showed significantly less interstitial fibrosis myocardial tissue between cardiomyocytes and lower expression of interleukin-6 (IL-6) compared to the untreated control group [108]. Thus, the antifibrotic effect of APL, which can prevent myocardial remodeling, was confirmed. This seems to be a noteworthy feature in the context of the remodeling properties of DOX, in the form of replacement of apoptotic cardiomyocytes with fibrous tissue, vacuolization and desquamation of surviving cardiomyocytes, and formation of cellular infiltrates as a side effect of DOX use.

DOX is known to severely disrupt cell homeostasis through multiple mechanisms described above. Any disruption in the cell environment carries the risk of activating regulated cell death (RCD) pathways. It has been examined that administration of [Pyr^1^]APL-13 after MI reduces cardiomyocyte apoptosis and myocardial remodeling. Male Wistar rats were divided into three groups: the first group consisted of healthy rats, the second group included animals after MI, and the third group included animals after MI with the implementation of [Pyr^1^]APL-13 treatment at a dose of 10 nmol/kg/day, ip for 5 days. Reverse transcription polymerase chain reaction (RT-PCR) analysis of the above groups showed that administration of [Pyr^1^]APL-13 increased anti-apoptotic Bcl-2 and decreased the expression of pro-apoptotic BAX family proteins and the apoptosis marker caspase-3. mRNA for the APL gene and APL receptor also increased. Hematoxylin and eosin (H&E) staining showed that APL-13 significantly reduced LV dilation, reversed changes in cardiomyocyte structure and shape, and reduced inflammation-related leukocyte infiltration, compared with control and myocardial infarction-induced groups [109].

## The apelinergic system in cardiovascular diseases (clinical studies)

Endogenous administration of APL increases CO and causes peripheral and coronary vasodilation in a study composed of 18 patients with HF, according to New York Heart Association (NYHA) class II to III and six patients undergoing diagnostic coronary angiography. In a randomized, double-blind study, patients with HF underwent acute administration of APL. Both systemic iv infusions of (Pyr^1^)APL-13 (30 to 300 nmol/min) and an intracoronary bolus of APL-36 (20 to 200 nmol) resulted in an increase in CO values and reduced peripheral vascular resistance. In addition, intrabrachial infusion of APL had the vasodilatory effect. These results show that administration of APL in the future may be protective in the treatment of patients suffering from chronic HF. APL may play an important role in the pathogenesis of chronic HF with EF < 35% [110].

In a cohort of 202 patients diagnosed with CHF, NYHA class forms I to IV, the concentration of plasma APL and NT-proBNP was measured. APL concentrations regardless of NYHA class were lower in patients with LVD than in healthy volunteers (3.76 vs. 0.85 ng/mL). It correlated positively with LVEF, right ventricle EF (RVEF), and peak of oxygen consumption (VO2) (*p* < 0.05). In contrast, N-terminal pro b-type natriuretic peptide (NT-proBNP) was higher in the study population than in the control group [111]. The results indicate that the apelinergic system is involved in the pathogenesis of heart failure.

### Mechanism of cardioprotective action of the apelinergic system in DOX-induced cardiotoxicity

There is limited investigation into how the expression of the apelinergic system and its modulations affect DOX-induced cardiotoxicity, which is usually the basis for treatment of the aforementioned cancer types.

### Preclinical research

Hamada et al. [112] in an acute mouse model of DOX-induced cardiotoxicity, investigated the apelinergic system. The mRNA expression levels of both APL and its receptor in the heart of C57Bl/6 J mice after DOX ip administration at a dose of 20 mg/kg were reduced on the first day after drug administration but reached normal levels on day 5. Angiotensinogen (AGT) and β-MHC, cardiac stress marker levels, were increased from days 1 to 5. In DOX-treated APJ knockout (APJ-KO) mice, ECHO examination indicated a reduction in LV internal diameter (LVID), FS, and heart rate (HR) compared to the saline group. The aforementioned parameters of p62 and microtubule-associated protein 1A/1B light chain 3 (LC3) in mutant hearts indicated the process of autophagy by increasing LC3 expression in the DOX-treated group of APJ-/- mice. Cell viability was studied by determining ATP levels in the H9c2 rat cardiomyoblast line. Pretreatment of these cells with APL resulted in attenuated DOX toxicity in H9c2/hAPJ cells compared to H9c2/mock cells [112]. Based on research conducted by Wang et al. APL-13 can reduce apoptosis, reduce the level of mortality of in vivo isolated DOX-treated rat primary cardiomyocytes, and increase the cell* s*urvival rate [49].

Saleme et al. [113] conducted a study on male nude mice subjected to transplantation of living cells of human lung cancer cells and iv administration of DOX in cumulative dose of 20 mg/kg. Cardiac dysfunction caused by DOX administration was observed during treatment. Subsequently, RNA sequencing was performed from cardiomyocytes isolated from the myocardium. Compared to the control group receiving green fluorescent protein (GFP), the DOX-treated group showed an increase in apoptosis-related genes, particularly the p53 gene and inhibition of cell survival pathways. In addition, a decrease in peroxisome proliferator-activated receptor gamma coactivator 1-alpha (PGC1A), transcriptional coactivator committed in regulating the genes involved in energy metabolism and biogenesis, was identified. Its activation is generally observed during cellular stress, formation of peroxisomes, and ROS formation [113, 114].

DOX treatment also decreased the expression levels of the APJ and APL itself which may affect fatty acid oxidation (FAO) caused by PGC1A, a regulator of lipid metabolism and fatty acid oxidation inductor. As a result, there was a reduction in oxidative phosphorylation in mitochondria. The decline in FAO associated with DOX-induced cardiotoxicity and diminished mRNA expression of APL and APJ, as well as PGC1A, may explain the dysfunction of cardiomyocyte contractility [113].

Chen et al. [115] investigated the effect of ELA on DOX cardiotoxicity in vitro on neonatal rat cardiomyocytes (NRC) and in vivo of male C57BL mice. Using calcein-AM/propidium iodide (AM/PI) staining and phalloidin staining, which stains live and dead cells, and LysoSensor and puncta mRFP-GFP-LC3 to measure autophagy flux in cells exposed to DOX, it was noted that DOX blocks autophagy flux, but ELA supply restores it. Blocking autophagy flux is a negative phenomenon, as maintaining homeostasis by removing degraded components determines normal cell function. DOX, however, causes accumulation of the resulting autolysosomes, disrupting the process of cellular equilibrium by inhibiting lysosome acidification. This phenomenon was explained by the discovery that DOX is responsible for inhibiting the EB transcription factor (TEEB) protein is responsible for lysosomal biogenesis and the normal process of autophagy. In an in vivo physical examination, measurement of body weight and heart weight provided information that DOX, through induction of cardiomyocyte atrophy, reduces heart weight of the mice tested. These parameters improved after ELA administration. Also, the ECHO examination showed a negative effect of DOX on cardiomyocytes in the context of deterioration of cardiac systolic parameters. However, ELA administration showed an improvement in the parameters of both LVEF and LVFS. All of this together provides information that ELA has beneficial effects on acute DIC in vitro and in vivo by improving TEEB-dependent autophagy flux and alleviating DOX-induced cardiac dysfunction [115].

In addition, Wang et al. [116] demonstrated the cardioprotective effect of ELA-11 in suppressing DOX-induced cardiotoxicity apoptosis in in vitro and in vivo models. It has been shown that ELA-11 can protect cardiomyocytes from apoptosis. It occurs through activation of ERK/MAPK and PI3K/AKT signaling pathways. TUNEL staining clearly showed that the number of apoptotic cells was increased in the DOX-induced group, but decreased after concomitant treatment with ELA-11. Interestingly, the effect of 4-oxo-6-((pyrimidin-2-ylthio) methyl)-4H-pyran-3-yl-4-nitrobenzoate (ML221), an APJ antagonist, on cardiomyocyte apoptosis after simultaneous administration of DOX and ELA-11 was also examined. ML221 was found to have an inhibitory effect on ELA-11 in the DOX model. This was consistent with the fact that phosphorylation of AKT, PI3K, and ERK proteins was reduced by ML221 compared to ELA-11. With the above observations, it was proven that the ELA-APJ axis is involved in regulation of cardiomyocyte apoptosis caused by DOX [116] (Table 2).

### Clinical studies

Bioinformatics analysis for exploring and identification of potential target genes of heart-specific microRNA (miR-130a-3p) in DIC by Hamid Ceylan included five samples from women with established DIC and ten from women undergoing cancer treatment but with undetected cardiotoxicity. The differentially expressed genes (DEGs) between HF due to DOX versus non-HF samples targeted by hsa-miR130a-3p were examined using microarray dataset GSE40447. The results revealed that downregulated DEGs targeted by miR-130a-3p was a gene named solute carrier family 8 sodium/calcium exchanger member 1 (SLC8A1). SLC8A1 is involved in downregulated APL signaling pathways in cardiomyocytes, regulated calcium reabsorption, cardiomyocyte contraction processes, and hypertrophic cardiomyopathy (HCM) and dilated cardiomyopathy (DCM). The results provide an opportunity to speculate that SLC8A1 is an important target gene of miR-130a-3p, and in turn miR-130a-3p contributes to the development of cardiac pathology. These outcomes confirm that the APJ system is a component that is impaired in DOX-induced cardiotoxicity through SLC8A1 [118].

## Conclusion

The studies presented in this article highlight the beneficial effects of the apelinergic system in DOX-induced cardiovascular pathologies. A study published by Hamada et al. showed that a single administration of DOX causes a significant decrease in mRNA expression of systemic and cardiac APL and APJ [112]. These findings reveal that DOX adversely affects APL and APJ expression, leading to cardiotoxicity in mouse hearts. These investigations were also confirmed in mice lacking the APJ gene, which showed greater susceptibility to cardiotoxicity, expressed by higher levels of myocardial necrosis markers and more prominent oxidative stress damage. Activation of survival pathways, protection from impaired ATP and calcium metabolism, and modulation of autophagy contribute to its cardioprotective effects [116].

The exploration of therapeutic strategies, including apelinergic analogs [98] and APJ agonists [132, 133], holds promise for treating DOX-induced cardiotoxicity. Combining endogenous APL [110] or its analogs with DOX therapy and exploring gene therapies targeting APL expression may further enhance protective mechanisms.

Overall, understanding and leveraging the cardioprotective nature of the apelinergic system present a compelling avenue for developing novel interventions against DOX-associated cardiovascular effects, urging further research for targeted clinical applications.

### Summary of state-of-the-art

In the scientific literature, the current state of knowledge in the context of the beneficial effects of the apelinergic system on the cardiovascular system is quite well documented. However, there is a paucity of information available on the benefits of this system in DOX-induced cardiotoxicity. This review aims to provide support for the hypothesis that the apelinergic system contributes to the reduction of anthracycline-induced cardiotoxicity. Animal studies, due to different models of cardiac injury, are difficult to compare, but their common denominator is improvement in cardiac systolic function as a result of activation of the apelinergic system.

This literature review was based on 132 references. During the collection of the material (2022–2023), the following databases were used: Embase, Google Scholar Medline, PubMed, ScienceDirect, Scopus, Web of Science. These databases were searched for information about the importance of the apelinergic system in doxorubicin-induced cardiotoxicity using the following phrases: “anthracycline cardiotoxicity, apelin, apelin receptor, apelinergic system, apelin, cancer, cardioprotective therapy, chemotherapy, elabela, doxorubicin, doxorubicin cardiotoxicity, oncotreatment” in a variety of combinations.

The information on the significance of the apelinergic system in doxorubicin-induced cardiotoxicity is preceded by a comprehensive introduction to the subject matter, which addresses the issue of cancer incidence and the adverse effects of chemotherapy, with a particular focus on the cardiotoxicity of DOX. In response to the necessity for cardioprotective therapies during cancer treatment, the review elucidates the characteristics of the apelinergic system in the context of its cardioprotective properties.

The present review is based on an analysis of literature data, including original papers and reviews. This analysis allowed the review to be divided into sections on the cardioprotective effects of the apelinergic system, in the context of both cell and animal studies and clinical trials. In order to ensure the greatest possible variety of research models, the preclinical studies were selected to include a wide range of different models. In contrast, the clinical trials were selected to include both pediatric and adult populations.

Given the increasing incidence of various types of cancer, whose treatment burdens the heart, the use of the apelinergic system seems to be a successful strategy. This review highlights its therapeutic potential based on the analysis of in vitro and in vivo studies.

## Abbreviations

ACC: Acetyl-CoA carboxylase
ACE: Angiotensin-converting enzyme
AGT: Angiotensinogen
AIF: Apoptosis-inducing factor
AM/PI: AM/propidium iodide
AMPK 5'AMP: activated protein kinase pathwaye
ANP: Atrial natriuretic peptide
ANT: Anthracyclines
APJ: Apelin receptor
APL: Apelin
a-SMA: Smooth muscle a-actin
ATG: Autophagy-related gene
ATP: Adenosine triphosphate
BAX Bcl-2: associated X protein
Bcl-1: Beclin-1
Bcl-2: B-Cell lymphoma 2
β-MHC: Myosin heavy chain
BNP: B-Type natriuretic peptide
CAD: Coronary artery disease
CaN/NFAT: Calcineurin/nuclear factor of activated T
CF: Cardiac fibroblasts
CHF: Congestive heart failure
CK-MB: Creatine kinase-myoglobin binding
CMRI: Magnetic resonance imaging
CO: Cardiac output
COX: Cytochrome c oxidase
CPK: Creatine phosphokinase
CTRCD: Cancer therapy-related cardiac dysfunction
Cx43: Connexin 43
DCM: Dilated cardiomyopathy
DEGs: Differentially expressed genes
DIC: Doxorubicin-induced cardiotoxicity
DNA: Deoxyribonucleic acid
DOX: Doxorubicin
DOXol: Doxorubicinol
DR4: Death receptor 4
DR5: Death receptor 5
DSBs: Double-strand breaks
ECG: Electrocardiography
ECHO: Echocardiography
EF: Ejection fraction
ELA: Elabela
eNOS: Endothelial nitric oxide synthase
ERK1/2: Extracellular signal-regulated kinase 1/2
FAO: Fatty acid oxidation
FIP200: RB1-inducible coiled-coil protein 1
FS: Fractional shortening
GFP: Green fluorescent protein
GLC4: Small cell lung cancer cell lines
GLS: Global longitudinal strain
GPCRs: G-Protein-coupled receptors
HCFs: Human cardiac fibroblasts
HCM: Hypertrophic cardiomyopathy
HF: Heart failure
H&E: Hematoxylin and eosin
HR: Heart rate
H/R: Hypoxia/reoxygenation
H2O2: Hydroperoxide
IC-OS: International Cardio-Oncology Society
IL-6: Interleukin-6
i-mtNOS: Mitochondrial-inducible nitric oxide synthase
iNOS: Inducible nitric oxide synthase
IP: Intraperitoneal injection
I/R: Ischemia/reperfusion injury
IRP1/IRP2: Iron regulatory proteins 1/iron regulatory proteins 2
ISO: Isoproterenol
IV: Intravenous injection
KO: Knockout
K562: Myeloblastic leukemia cell lines
LAD: Left anterior descending artery
LC3: Microtubule-associated protein 1A/1B light chain 3
LC3-II: Phosphatidylethanolamine conjugate
LDH: Lactate dehydrogenase
LO: Lipid peroxidation
LV: Left ventricle
LVD: Left ventricular dysfunction
LVEDP: Left ventricular end-diastolic pressure
LVEF: Left ventricular ejection fraction
LVESD: Left ventricular end-systolic diameter
LVESP: Left ventricular end-systolic pressure
LVID: Left ventricular internal diameter
LVSD: Left ventricular systolic dysfunction
MABP: Mean arterial blood pressure
MAP: Mitogen-activated protein kinase
mCoQ10: Mitochondrial coenzyme 10
MDA: Malondialdehyde
MI: Myocardial infarction
MLC2: Myosin light chain-2
ML221: 4-oxo-6-((pyrimidin-2-ylthio)methyl)-4H-pyran-3-yl 4-nitrobenzoate
mPTP: Mitochondrial permeability transition pore
mRFTs: Mitochondrial reactive oxygen species
mTOR: Mechanistic/mammalian target of rapamycin
MUGA: Multigated acquisition scanning
NADH: Nicotinamide adenine dinucleotide + hydrogen
NCX: Na +-Ca2 + exchanger
NFAT: Nuclear factor of activated T cells
NF-κΒ: Nuclear factor-κB
nNOS: Neuronal nitric oxide synthase
NO: Nitric oxide
NOS: Nitric oxide synthase
NRC: Neonatal rat cardiomyocytes
NRVMs: Neonatal rat ventricular myocytes
NT-proBNP: N-Terminal pro b-type natriuretic peptide
NYHA: New York Heart Association
•OH: Hydroxyl radical
ONOO: Peroxynitrite
PGC1A: Proliferator-activated receptor gamma coactivator 1-alpha
PI3K: Phosphoinositide 3-kinase
PI3K/AKT: Phosphatidylinositol-3-kinase/protein kinase B survival pathway
PKB: Protein kinase B
PKC: Protein kinase C
p-PI3KC3: Phospho-PI3 kinase class III
RAAS: Renin-angiotensin-aldosterone system
RCD: Regulated death cell pathways
ROS: Reactive oxygen species
RT-PCR: Reverse transcription polymerase chain reaction
RVEF: Right ventricle ejection fraction
RYR: Ryanodine receptor Ca2 + -releasing channel
SD: SD Sprague-Dawley rats
SERCA: Sarcoplasmic/endoplasmic reticulum Ca^2^ + -ATPase
SLC8A1: Solute carrier family 8 sodium/calcium exchanger member 1
SOD: Superoxide dismutase
SSB: Single-strand breaks
SV: Stroke volume
TAC: Transverse aortic constriction
TEEB: EB transcription factor protein
TfR: Transferrin receptor
TGF-β: Transforming growth factor-b β
TGF-β1: Transforming growth factor β1
TNF-α: Tumor necrosis factor α
TNFR1: Fas receptor and tumor necrosis factor receptor 1
TOP2β: Topoisomerase II-beta
TOP: Topoisomerases
TUNEL: Terminal deoxynucleotidyl transferase dUTP nick end labeling
ULK1: Unc-51-like kinase 1
WT: Wild type
5-HT: Serotonin
